# The job creep-quiet quitting nexus revisited: servant leadership as a moderator in the manufacturing sector

**DOI:** 10.3389/fpsyg.2026.1895178

**Published:** 2026-08-06

**Authors:** Mehmet Ali Canbolat, Esra Yıldız, Nezahat Koçyiğit, Aykut Bedük, Enver Karakışla

**Affiliations:** 1 Department of Management and Organization, Karamanoğlu Mehmetbey University, Karaman, Türkiye; 2 Department of Accounting and Taxation, Karamanoğlu Mehmetbey University, Karaman, Türkiye; 3 Department of Management Information Systems, Necmettin Erbakan University, Konya, Türkiye; 4 Department of Management and Organization, Selçuk University, Konya, Türkiye; 5 Department of Finance, Banking and Insurance, Karamanoğlu Mehmetbey University, Karaman, Türkiye

**Keywords:** job creep, manufacturing sector, moderating role, quiet quitting, servant leadership

## Abstract

This study aims to examine the organizational and managerial factors influencing employees’ quiet quitting behavior. In this context, the study investigates the effect of job creep on quiet quitting and the moderating role of servant leadership in this relationship. A quantitative research design was adopted, and data were collected via a survey of 386 employees working at manufacturing firms in the Karaman Organized Industrial Zone. Using SPSS, the collected data were analyzed for reliability and correlation, and confirmatory factor analysis was conducted; hypotheses were tested using the Hayes Process Macro (Model 1). The findings indicate that job creep has a significant and positive effect on quiet quitting. Although servant leadership did not have a direct significant effect, it significantly moderated the relationship between job creep and quiet quitting. The findings indicate that an increase in employees’ perceptions of their workload leads to increased quiet quitting behavior and highlight the importance of leadership approaches that balance employee expectations with workplace demands.

## 1 Introduction

In contemporary working life, employees are increasingly expected not only to fulfill their official duties but also to assume additional responsibilities beyond their job descriptions. While such off-role behaviors are often viewed positively in terms of organizational effectiveness and performance, over time, the institutionalization of these behaviors can lead employees to perceive voluntary contributions as an implicit obligation. This situation, conceptualized as job creep, can lead employees to develop perceptions of excessive workload, role ambiguity, decreased autonomy, and increased psychological pressure (Van Dyne and Ellis, 2004). Employees can experience psychological strain, especially in work environments where workload and performance expectations increase and work-life boundaries become increasingly blurred. This can lead to negative consequences such as emotional burnout, alienation from work, and withdrawal behaviors. Quiet quitting is considered one of the current forms of withdrawal behaviors and refers to employees avoiding effort and voluntary contributions outside their roles by only fulfilling the responsibilities included in their job descriptions (Çimen and Yılmaz, 2023). Employees exhibiting quiet quitting do not physically leave the organization; however, their psychological commitment and voluntary contributions to the organization significantly decrease. The theoretical basis of this research is the Job Demands–Resources (JD-R) theory. According to JD-R, when job demands exceed the physical and psychological resources available to employees, over time, it leads to burnout, alienation from work, and withdrawal behaviors (Demerouti et al., 2001). Job demands refer to working conditions that require continuous physical, cognitive, or emotional effort, while job resources encompass managerial, social, and organizational elements that facilitate employees’ ability to cope with these demands. In this context, job overflow can be considered a significant job demand, leading employees to assume additional responsibilities beyond their formal job descriptions. In contrast, quiet quitting can emerge as a behavioral coping strategy aimed at conserving employees’ limited resources. In this process, servant leadership is considered a crucial resource that can influence employees’ assessment of and responses to increasing work demands. In this context, leadership style is regarded as a significant determinant of employees’ responses to challenging working conditions. The servant leadership approach prioritizes employees’ needs, supports their development, fosters empathy, and builds trust-based relationships (Greenleaf, 1977; Spears, 2005). Because of these characteristics, servant leadership is believed to influence employees’ attitudes and behaviors when faced with increasing workload and role pressure. The manufacturing sector offers a suitable research context for examining the relationship between job burnout and quiet quitting due to its intensive production processes, high performance expectations, and demanding working conditions. In this sector, employees may face long working hours, constant performance pressure, and increasing task demands. Therefore, identifying the organizational dynamics that give rise to quiet quitting behavior is important both for supporting employee well-being and for developing sustainable human resources practices for businesses. Although quiet quitting, servant leadership, and job creep have been examined separately in the literature with respect to related variables, studies examining the direct relationship between job creep and quiet quitting are limited. Furthermore, empirical evidence regarding the moderating role of servant leadership in this relationship is insufficient. Accordingly, this study aims to examine the effect of job creep on quiet quitting among employees in the manufacturing sector in Türkiye and to test the moderating role of servant leadership in this relationship. The research findings are expected to contribute theoretically and practically to the literature on quiet quitting by integrating the leadership perspective within the framework of Job Demands-Resources Theory. In this study, job creep is considered a job demand that causes employees to take on responsibilities outside their core duties. In contrast, quiet quitting is considered a behavioral withdrawal response that employees develop to protect their existing resources when facing increasing job demands. Job Demands-Resources Theory also suggests that the resources available for work are used to meet increasing job demands.

## 2 Theoretical framework

This section reviews the theoretical relationships between job creep, quiet quitting, and servant leadership and presents the study hypotheses.

### 2.1 Job creep

One of the concepts that has been positively received in the field of organizational behavior in recent years is organizational citizenship behavior (OCB) (Organ, 1988; Podsakoff et al., 2000). Defined as voluntary behaviors that have the potential to increase organizational effectiveness beyond formal job descriptions, OCB includes five dimensions: altruism, civic virtue, courtesy, conscientiousness, and sportsmanship (Organ, 1988). OCB represents activities such as helping other employees in the organization, being involved in future developments, avoiding behaviors that may cause tension, punctuality, and careful use of tea/coffee and meal breaks (Podsakoff and Mackenzie, 1994). Therefore, the type of behavior that are most desired by organizations in high-performing employees is voluntary, enjoyful, communicative employee behavior that aligns with the goals of the organization (Akgemci and Koçyiğit, 2013). However, OCB’s becoming an expectation over time may increase workload and result in psychological reactions. This situation presents a perspective contrary to the traditionally positive view of voluntary behavior (VBD), indicating that VBD can, in some cases, lead to a negative situation called “job creep.” Job creep is defined as the process by which an individual's job description gradually expands, making voluntary behaviors compulsory. It is argued that this situation can threaten individuals' perceived autonomy, cause stress and burnout, and that the resulting tension can trigger motivation to regain their freedom (Van Dyne and Ellis, 2004).Indeed, Reactance Theory suggests that individuals experience psychological tension when they feel their behavioral freedom is threatened (Brehm, 1966). The concept of “job creep” was first defined by Van Dyne and Ellis (2004). It refers to the process where behaviors voluntarily exhibited by employees once become consistently expected and are continued by the employee (Canbolat, 2024). It is argued that the psychological pressure on employees who do not perform the expected over-the-job behaviors is not immediately noticeable, making job creep an ongoing process. Since extra-role behaviors that are not formally defined are not rewarded by employers or managers, the pressure to “get more for less” leads to job creep among employees. When employees’ expanding workloads are not compensated with salary or promotion, this results in a violation of the reciprocity norm, i.e., the psychological contract (Van Dyne and Ellis, 2004). In summary, since the tasks that employees initially performed voluntarily are not considered “extra,” they are perceived as in-role behavior. For example, it is stated that a manager may interfere with employees’ leave requests so that they stay late to do extra work on a project (Bolino et al., 2013). It is noted that the negative feelings arising from this can lead to counterproductive behaviors, withdrawal, and work–family conflict (Spector et al., 2006).

### 2.2 Quiet quitting

The concept of quiet quitting first emerged in 2009. It gained prominence on social media in 2021 with the “Tang Ping” movement in China, and again in 2022 with a TikTok video. The term is also referred to in the literature as “Silent Resignation,” “Silent Giving Up,” and “Silent Exit. The concept was first used in a video published on TikTok and YouTube in March 2022 by Bryan Creely, a recruitment specialist and career coach (Daugherty, 2022). In 2022, the concept became widespread.

It was made visible again in a TikTok video by a young American engineer who said, “Work is not your life.” This video prompted everyone in the workforce to question their working conditions and led to the spread of the idea of quiet quitting (Güler, 2023). Quiet quitting is defined as individuals not taking responsibility outside of their primary duties, giving up on voluntary work, prioritizing their private lives by not answering work-related phone calls and emails after working hours, and not putting in much effort while fulfilling their duties (Çimen and Yılmaz, 2023).

The emergence of quiet quitting behavior is significantly shaped by employers’ unlawful practices compelling employees to work well beyond legally prescribed hours and engage in excessive overtime. Such conditions frequently precipitate burnout among employees, ultimately driving them toward quiet quitting. Particularly following the COVID-19 pandemic, changing employee expectations regarding work–life balance, flexible working arrangements, and psychological well-being have contributed to the growing visibility of quiet quitting behaviors among employees (Avcı, 2023, p. 923). Within the organizational behavior literature, the concept of quiet quitting is theoretically grounded in Conservation of Resources Theory, Self-Determination Theory, and Social Exchange Theory. According to Conservation of Resources Theory, employees endeavor to preserve and enhance their personal resources—such as effort, skills, and knowledge—while performing their duties, as these resources are instrumental in securing returns, including wages, bonuses, rewards, and improved working conditions. When employees fail to obtain the anticipated returns from the organization, they may resort to quiet quitting as a defensive mechanism to prevent further depletion of their resources (Arar et al., 2023; Karavelioğlu et al., 2023). From the perspective of Self-Determination Theory, individual behavior is fundamentally driven by the needs for autonomy, competence, and relatedness (Deci et al., 2017). In this regard, the fulfillment of employees’ basic psychological needs is essential to mitigate the likelihood of quiet quitting.

Employees who receive adequate managerial support are more inclined to engage in constructive and value-adding activities for the organization. Finally, Social Exchange Theory posits that relationships between parties are inherently characterized by mutual interdependence. Accordingly, disruptions in the reciprocity between employee and employer, or the failure to meet employee expectations, may lead individuals to restrict their contributions to the bare minimum required by their roles, thereby manifesting quiet quitting behavior (Karavelioğlu et al., 2023).Empirical studies have identified a range of antecedents contributing to quiet quitting, including the absence of clear career pathways within the organization, poor work–life balance, inadequate remuneration, job descriptions, extended working hours, excessive organizational performance expectations, lack of recognition for performance, experiences of disrespect, diminished professional fulfilment, erosion of the perceived meaning of work, misalignment between individual and organizational goals, deficiencies in communication and trust, lack of organizational belonging, feelings of being undervalued, and managerial inconsistency (Formica and Sfodera, 2022; Kaplan, 2022; Youthall, 2022; Çimen and Yılmaz, 2023).

During the quiet quitting process, employees tend to display reduced engagement, lower discretionary effort, psychological withdrawal, and declining participation in organizational activities (Hetler, 2022). Employees exhibiting quiet quitting tendencies generally limit their contributions to formally required tasks and avoid extra-role behaviors.

Quiet quitting engenders a wide array of both positive and negative consequences at individual and organizational levels within enterprises. As a consequence of quiet quitting, employees fail to fully realize their potential, leading not only to diminished performance but also to adverse implications for their career trajectories (Saygılı and Avcı, 2023). In order to mitigate the detrimental organizational impacts of this phenomenon, emphasis should be placed on preventive measures such as the refinement of managerial processes, the establishment of transparent and open communication policies, and the adoption of a supportive management approach. Moreover, identifying the underlying causes of this behavior; fostering awareness among employees; maintaining continuous and meaningful communication; demonstrating empathy; and implementing practices that reinforce employees’ sense of value and empowerment are critical interventions that managers can undertake. Such measures may, in turn, reduce the likelihood of employees exhibiting quiet quitting behavior.

### 2.3 Servant leadership

Servant leadership was first conceptualized by Robert K. Greenleaf as an approach grounded in prioritizing followers’ needs, development, and well-being. Unlike traditional leadership approaches that primarily emphasize authority and organizational outcomes, servant leadership focuses on serving employees, fostering trust, and supporting personal growth. Servant leaders are characterized by behaviors such as empathy, active listening, ethical conduct, emotional support, and commitment to employee development (Greenleaf, 1977; Spears, 2005; Van Dierendonck, 2011).

In organizational settings, servant leadership is considered an employee-centered leadership style that strengthens interpersonal relationships and promotes psychological well-being. Employees who perceive their leaders as supportive and caring are more likely to experience higher levels of motivation, organizational commitment, and job satisfaction. Moreover, servant leaders contribute to the creation of a positive organizational climate through open communication, fairness, and empowerment practices (Liden et al., 2008; Van Dierendonck, 2011).

Within the context of increasing workload and job-related stress, servant leadership may play an important role in shaping employees’ reactions to adverse working conditions. Since servant leaders tend to prioritize employees’ emotional and professional needs, they may help reduce negative outcomes such as disengagement, burnout, and withdrawal behaviors (Liden et al., 2015; Özbezek, 2022). Therefore, servant leadership is considered a relevant organizational resource for understanding employees’ quiet quitting tendencies under conditions of job creep.

However, recent studies suggest that servant leadership may not always function as a protective mechanism in stressful work environments. In certain situations, employees may perceive supportive leadership behaviors as an implicit expectation to assume additional responsibilities, potentially intensifying emotional exhaustion and withdrawal tendencies (Bolino et al., 2013; Güler, 2025). Accordingly, examining the moderating role of servant leadership in the relationship between job creep and quiet quitting may provide important theoretical and practical insights.

While servant leadership is often associated with positive outcomes such as high organizational commitment and job performance, current literature suggests that the effects of this leadership style may not always be beneficial and may have a “dark side” under certain circumstances (Pierce and Aguinis, 2013; Camm, 2019; Kessler, 2019; Gao and Liu, 2023).

The approaches of servant leaders, who support, empathize with, and prioritize the needs of their employees, foster a strong sense of moral obligation and psychological indebtedness toward the leader and the organization. While this sense of indebtedness is normally considered positive, in labor-intensive environments where job demands are already high, it can unintentionally encourage employees to take on additional responsibilities beyond their formal job descriptions (job creep) and make role expansion a necessary norm (Canbolat, 2024). From the perspective of Job Demands-Resources (JD-R) theory (Bakker and Demerouti, 2007), although servant leadership theoretically functions as a ‘job resource,’ when job-related demands are excessively high, it loses this resource role, increasing the pressure on employees to internalize the extra workload in the name of loyalty to the leader, escalating organizational tension, and potentially reducing organizational commitment (Palumbo, 2016). Consequently, instead of mitigating the negative effects of increasing job demands, servant leadership can paradoxically reinforce the relationship between increased job demands and quiet quitting—a passive withdrawal behavior—by normalizing out-of-role expectations and thus leading to faster depletion of employees’ limited resources (Canavesi and Minelli, 2022).

### 2.4 The relationship between job creep, quiet quitting and servant leadership

A substantial body of research has examined the interrelations among job creep, quiet quitting, and servant leadership. For instance, Zhang and Rodrigue (2023), in their study focusing on working mothers, found that mothers who were unable to obtain maternity leave exhibited higher levels of quiet quitting behavior compared to those who had access to maternity leave and childcare support in the workplace. Moreover, mothers who perceived insufficient organizational support and lack of recognition were more likely to disengage quietly from their work. Accordingly, employers who provide clear career advancement opportunities and adopt transparent policies that address the expectations of working mothers may mitigate levels of dissatisfaction that contribute to quiet quitting behavior. In a study conducted by Kepenekci et al. (2024) with teachers, it was determined that quiet quitting did not significantly differ according to gender, school level, or subject area. The authors therefore emphasized that enhancing teachers’ job satisfaction and motivation, alongside improving working conditions and the organizational environment, constitute a more effective strategy for preventing quiet quitting. Similarly, Altun et al. (2024), in their study of employees in the patient services unit of a university hospital, identified a high prevalence of quiet quitting behavior. Notably, employees working more than 45 h per week, employees with 3–5 years’ tenure, and female employees demonstrated higher levels of such behavior than their counterparts. Another study of healthcare professionals examined the relationships among work–life balance, burnout, and quiet quitting, finding that individuals with high work–life balance were less likely to engage in quiet quitting, even when experiencing burnout. This finding suggests that policies grounded in work–life balance may serve as an effective mechanism for reducing tendencies toward quiet quitting (Bakır and Özcan, 2025). Furthermore, research involving bank employees demonstrated a significant relationship between quiet quitting and leader–member exchange, indicating that higher-quality leader–member interactions may reduce the likelihood of quiet quitting behavior (Örücü and Hasırcı, 2024). With regard to servant leadership, a review of the literature indicates that Akgemci et al. (2019) investigated the impact of servant leadership behaviors on employee voice, concluding that such leadership behaviors exert a significant influence on the voice behavior of research assistants. Conversely, a study conducted with employees in the food and tourism sectors found that servant leadership did not exhibit a moderating effect on the relationship between burnout and organizational citizenship behavior (Güler, 2025). Similarly, research involving employees in manufacturing enterprises concluded that servant leadership did not play a moderating role in the relationship between job satisfaction and task performance (Yücekaya, 2021).

**Figure 1:**
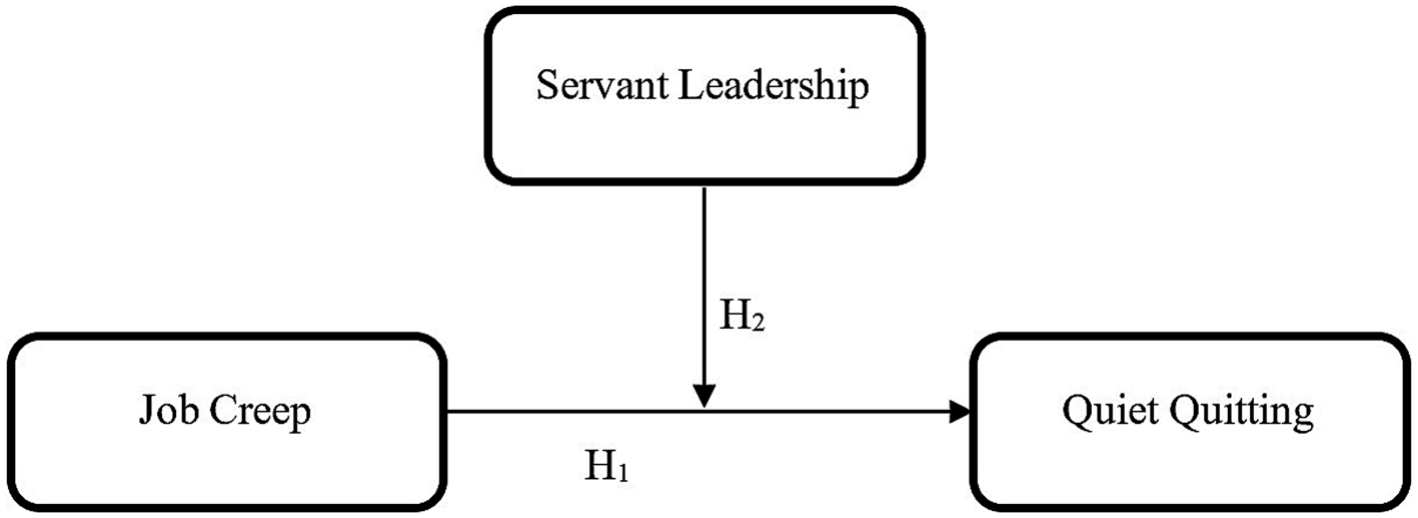
Research model.

Despite the growing body of research addressing these constructs individually or in partial combinations, no study has been identified that directly examines the interrelationship between job creep, quiet quitting, and servant leadership in a comprehensive manner. In light of these considerations, the research model developed within the present study is illustrated in Figure 1.

The hypotheses proposed in Figure 1 are as follows:

*H*_1_: Job creep exerts a positive effect on quiet quitting.

Based on the JD-R framework, excessive workload and expanding role expectations may increase employees’ tendency toward psychological withdrawal. In this context, leadership style may shape how employees respond to these job demands.

*H*_2_: Servant leadership moderates the effect of job creep on quiet quitting.

## 3 Materials and methods

This section presents the population and sample for the quantitative research, the data collection tools, and the findings from the data analyses. Data were analyzed using IBM SPSS and AMOS software for reliability, correlation, confirmatory factor analysis, and moderation analysis. Finally, ethical approval to determine the suitability of the research with respect to scientific ethics was obtained from the ethics committee of a state university in Türkiye on January 22, 2026 (approval number E-75732670-100-310909).

The study population comprises employees of manufacturing enterprises located in the Karaman Organized Industrial Zone in Turkiye. According to available data, the Karaman Organized Industrial Zone employs approximately 11,090 individuals (Karaman OSB, 2026). The selection of Karaman as the research population is grounded in its economic significance, as the province accounts for approximately 0.30% of Türkiye’s total Gross Domestic Product (GDP).

Furthermore, according to data from the Istanbul Chamber of Industry, a total of four companies from Karaman were listed among the top 1,000 industrial enterprises in 2024 (ISO, 2026). This region exemplifies a typical labor-intensive manufacturing sector in developing countries, where job creep is prevalent and readily apparent. The sample size was calculated to be 372 participants based on a 95% confidence level and a 5% margin of error, using standard sample size estimation formulas. Accordingly, the researchers obtained a total of 398 data collection forms between February and March 2026. Statistical analyses were conducted on data from 386 valid questionnaires after excluding incomplete or invalid responses.

Data were collected using a structured cross-sectional questionnaire consisting of four distinct sections. The first section contains 5 questions collecting demographic information; the second contains the job creep scale; the third contains the quiet quitting scale; and the fourth contains the servant leadership scale. The job creep scale was developed in Turkish by Canbolat (2024) as a 13-item scale and consists of 3 dimensions: additional workload (1, 2, 3, 4, 5, 6), helpfulness (7, 8, 9, 10), and withdrawal (11, 12, 13). The quiet quitting scale was developed by Jehle and Schmitz (2007) as a 5-item, single-dimension scale, and its Turkish adaptation was carried out by Seçer (2011). The servant leadership scale was first developed by Liden et al. (2008) as a 28-item, 7-dimension scale. However, because the scale contained many items, Liden et al. (2015) developed a short form consisting of 7 items and a single dimension. The Turkish adaptation of the scale was carried out by Kılıç and Aydın (2016). The scales were used with a five-point Likert type ranging from 1-Strongly disagree to 5-Strongly agree.

The principle of voluntary participation was observed during data collection. Before the survey, each participant was informed about the content of the study and their consent was verbally confirmed; then they were asked to approve the informed consent statement in the introductory section of the survey form, which read, “I agree to participate in the research voluntarily.” As a result of this consent mechanism, informed consent from all study participants was documented in accordance with academic and ethical standards. The main reason for choosing a cross-sectional design in this study is its time- and cost-effectiveness, which allows data to be collected from a large population of workers in manufacturing businesses within the Karaman Organized Industrial Zone in a short period while remaining within the predetermined margin of error and confidence limits. In addition, the cross-sectional approach is suitable for identifying, at a single point in time, the current state of dynamic organizational variables such as job creep, quiet quitting, and servant leadership, and the regulatory relationships among them.

## 4 Results

Initially, frequency analysis was employed in the study. According to the findings, the mean age of the participants was 34.04 years (min: 18, max: 66). Other demographic findings are presented in Table 1.

**Table 1:** Demographic characteristics of the participants (*n* = 386).

Variables	%	Frequency
Gender		
Female	17.4	67
Male	82.6	319
Educational Status		
Primary School	24.1	93
High School	42.7	165
Associate Degree	17.1	66
Bachelor’s Degree	14.2	55
Post-Graduate Degree	1.8	7
Marital Status		
Married	55.4	214
Single	44.6	172
Work Experience		
0–1 years	12.2	47
2–5 years	28.8	111
6–10 years	28.0	108
11–20 years	19.2	74
Over 20 years	11.9	46

According to Table 1, the majority of participants are male (82.6%), are high school graduates (42.7%), are married (55.4%), and have between 2 and 5 years of work experience (28.8%). Following these descriptive findings, reliability and correlation analyses were conducted to assess the internal consistency of the scales and examine the relationships among the variables. The results are presented in Table 2.

**Table 2:** Findings of correlation, Cronbach’s alpha, mean, and standard deviation analyses.

Variables	Mean	SD	1	2	3	4	5	6
1-Job Creep	3.54	0.751	(0.858)					
2-Additional Workload	3.49	0.992	0.906^**^	(0.872)				
3-Helpfulness	4.05	0.871	0.583^**^	0.336^**^	(0.827)			
4-Withdrawal	2.98	1.151	0.679^**^	0.498^**^	0.061	(0.807)		
5-Quiet quitting	3.06	1.065	0.585^**^	0.502^**^	0.117^*^	0.672^**^	(0.856)	
6-Servant Leadership	3.71	0.945	0.052	−0.011	0.215^**^	−0.051	−0.018	(0.897)

**Correlation is significant at the 0.01 level (2-tailed).

*The correlation is significant at the 0.05 level (2-tailed).

The values presented in parentheses represent the Cronbach’s Alpha coefficients of the variables. SD = Standard Deviation.

Table 2 shows that, according to Cronbach’s alpha analysis, each scale and its sub-dimensions exhibit high reliability. The means of the scales and sub-dimensions ranged from 2.98 to 4.05. Correlation analysis revealed a moderately positive, significant relationship between quiet quitting and the job creep scale, the additional workload sub-dimension, and the withdrawal sub-dimension. The relationship between quiet quitting and the helpfulness sub-dimension was positive and statistically significant, but weak. Furthermore, a statistically significant, positive, but weak relationship was observed between servant leadership and the helpfulness sub-dimension of job creep. No relationship was found between servant leadership and the other sub-dimensions and scales (*p* > 0.05).

**Figure 2:**
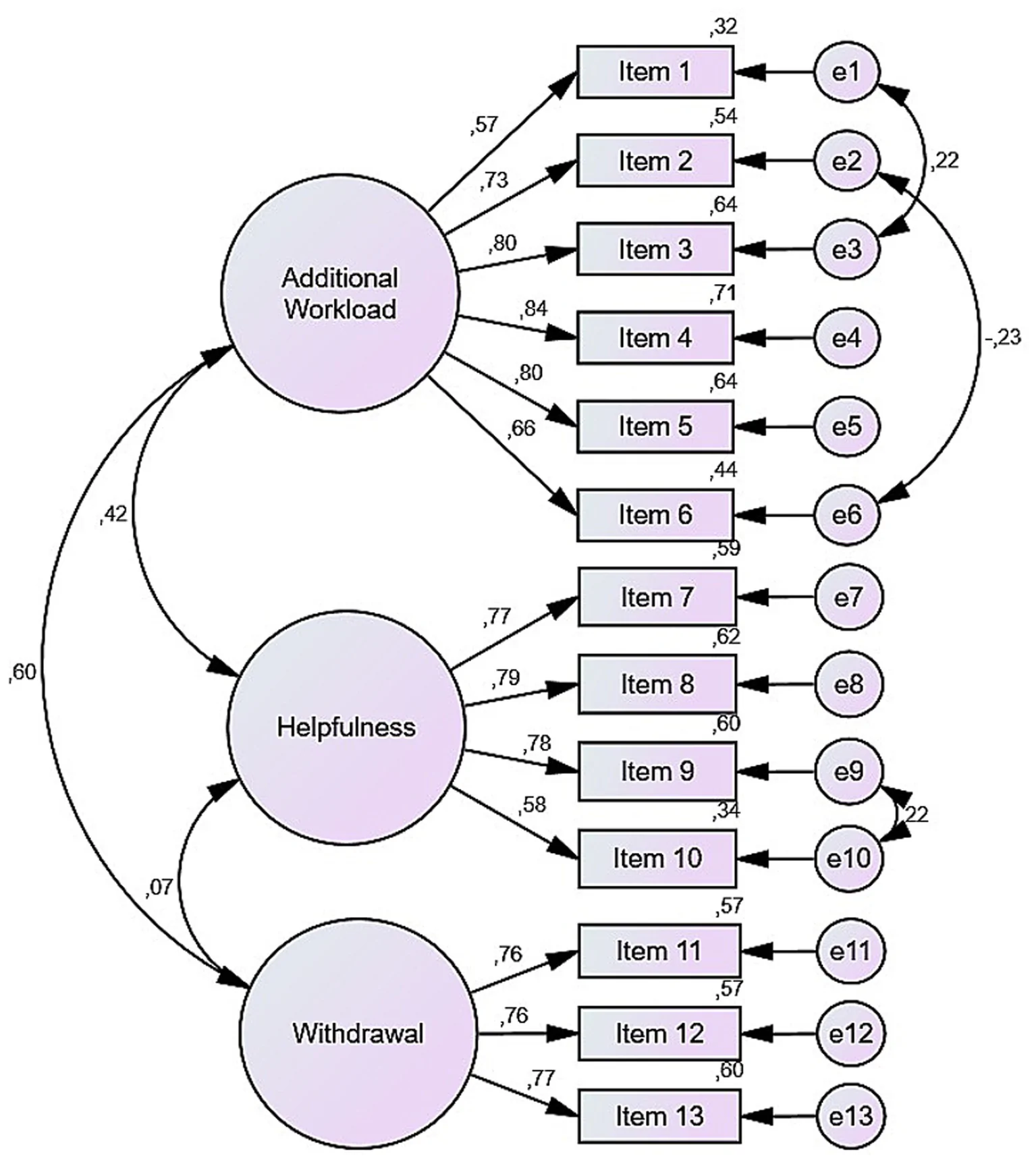
First-order multi-factor confirmatory factor analysis model of the job creep scale.

Harman’s single-factor test was conducted to assess common method bias. The results indicated that a single factor did not account for the majority of the variance, suggesting that common method bias was not a serious concern in the study. After this stage, confirmatory factor analysis (CFA) was performed to test the construct validity of the scales using the obtained data. The first-order multifactorial CFA model applied to the job creep scale is shown in Figure 2.

Accordingly, the standardized factor loadings of the items in the scale range from 0.57 to 0.84. The fact that these coefficients exceed the threshold of 0.40 indicates that the items exhibit a satisfactory level of fit with their respective sub-dimensions (Başar, 2016).

**Figure 3:**
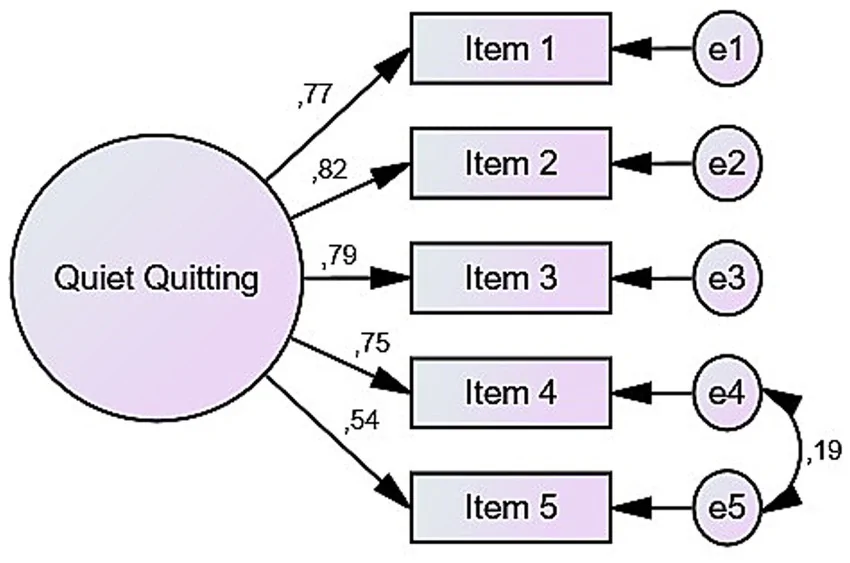
First-order multi-factor confirmatory factor analysis model of the quiet quitting scale.

Furthermore, the correlation coefficients among the dimensions vary between 0.07 and 0.60, suggesting the absence of multicollinearity issues among the dimensions. Figure 3 presents the single-factor confirmatory factor analysis (CFA) model used to assess the construct validity of the quiet quitting scale.

Accordingly, the standardized factor loadings of the items within the scale range from 0.54 to 0.82.

**Figure 4:**
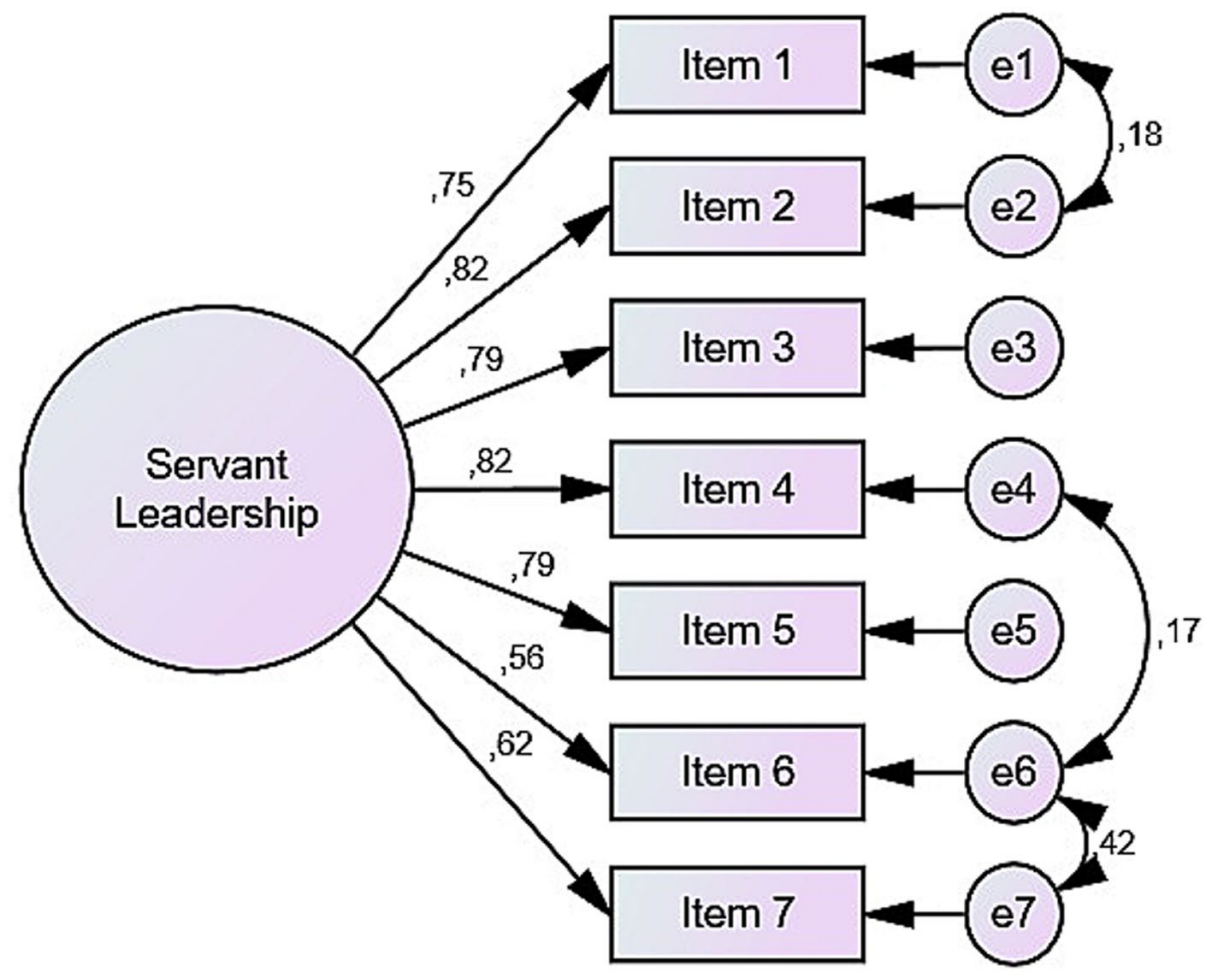
Single-factor confirmatory factor analysis model of the servant leadership scale accordingly, the standardized factor loadings of the items on the scale range from 0.56 to 0.82.

Figure 4 presents the single-factor confirmatory factor analysis (CFA) model used to assess the construct validity of the servant leadership scale.

Table 3 also presents the CFA models’ fit indices and the reference thresholds for “good fit” and “acceptable fit.”

**Table 3:** Fit indices of the Confirmatory Factor Analysis (CFA) models for the scales.

Index	Job creep scale	Quiet quitting scale	Servant leadership	Good fit	Acceptable fit
*X*^2^/df	2.152	1.799	3.354	<3	3 < (*X*^2^/df) < 5
RMSEA	0.055	0.046	0.078	<0.05	<0.08
SRMR	0.044	0.014	0.031	<0.05	<0.08
CFI	0.970	0.996	0.983	>0.95	>0.90
NFI	0.946	0.991	0.976	>0.95	>0.90
GFI	0.951	0.992	0.972	>0.95	>0.90
AGFI	0.925	0.971	0.930	>0.95	>0.90

*X*^2^/df = Chi-Square/Degree of Freedom, RMSEA = Root Mean Square Error of Approximation, SRMR = Standardized Root Mean Square Residual, CFI = Comparative Fit Index, NFI = Normed Fit Index, GFI = Goodness-of-Fit Index, AGFI = Adjusted Goodness-of-Fit-Index. Source: Schermelleh-Engel et al. (2003), Gürbüz (2021).

According to Table 3, RMSEA, NFI, and AGFI fit indices of the job creep scale fall within acceptable thresholds, while the remaining fit indices indicate a good fit. The quiet quitting scale, on the other hand, exhibits a good fit across all fit indices. Finally, an examination of the servant leadership scale reveals that the *χ*^2^/df, RMSEA, and AGFI indices are within acceptable limits, while the remaining indices indicate a good fit.

In light of these findings, it can be concluded that the construct validity of all scales has been satisfactorily established. When evaluated collectively, the results indicate that the scales employed in the study are empirically supported by the data. Following this stage, hypothesis testing was conducted.

For the purpose of testing the hypotheses, the Process Macro developed by Hayes (2018), which operates as an add-on to IBM SPSS, was utilized. The model employed in the analysis corresponds to Model 1. The findings are presented in Table 4.

**Table 4:** Regression analysis results indicating the moderating effect (*n* = 386).

Variables	*b*	C. I.	S. E.	*t*
Constant	3.058*	[2.97, 3.14]	0.044	69.94
Job Creep (X)	0.823*	[0.71, 0.94]	0.058	14.08
Servant Leadership (W)	−0.062	[−0.15, 0.03]	0.047	−1.33
X. W	0.129**	[0.03, 0.23]	0.053	2.45

*R* = 0.59, *R*^2^ = 0.355; *p < 0.001, **p < 0.05. S. E., standard error; C. I., confidence interval. b, unstandardized beta coefficient.

**Figure 5:**
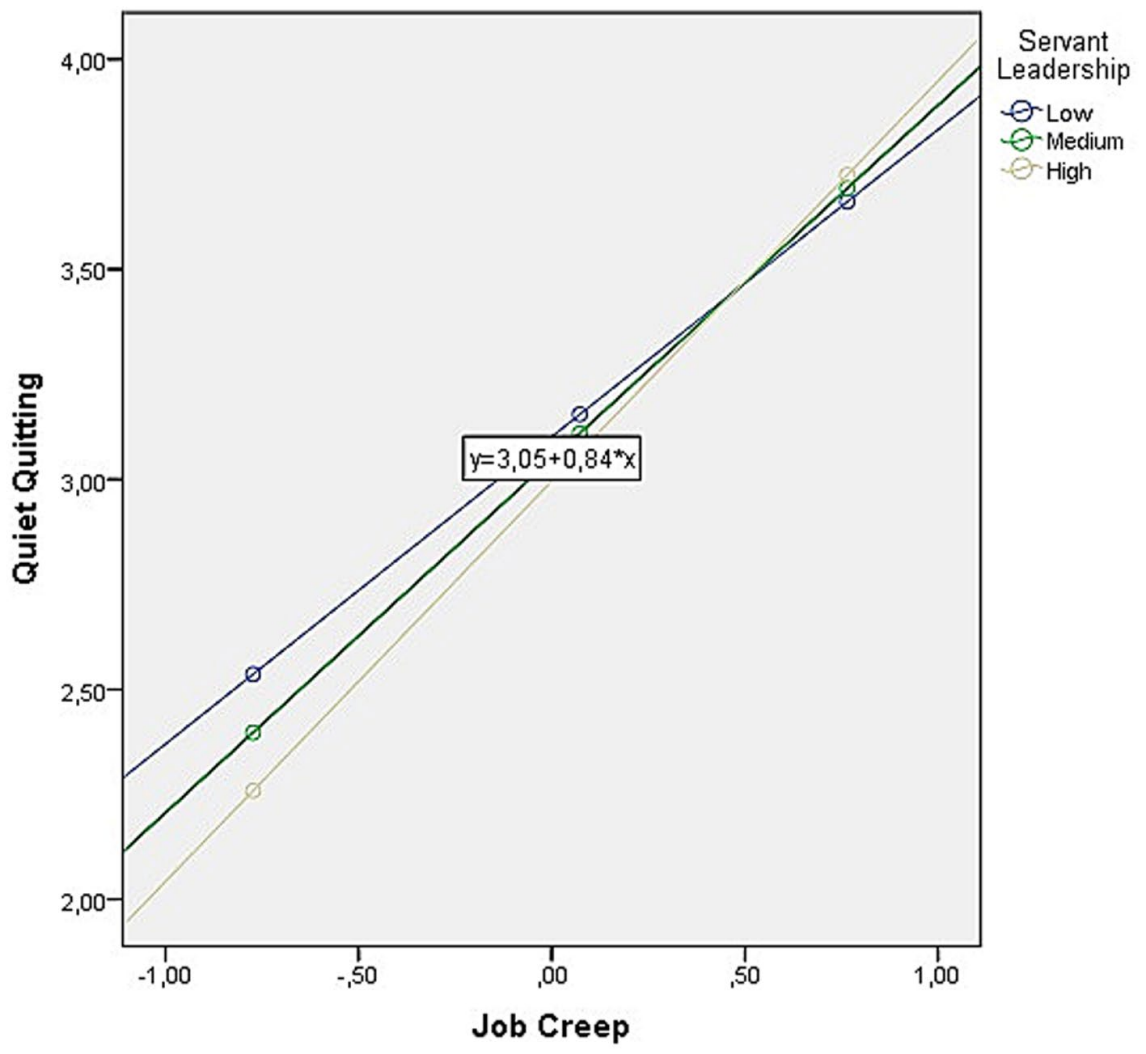
Moderation effect graph.

The results presented in Table 4 indicate that all predictor variables included in the regression analysis explain approximately 36% of the variance in quiet quitting (*R*^2^ = 0.355). Firstly, job creep has a statistically significant and positive effect on quiet quitting (*b* = 0.82, *p* < 0.001). Accordingly, Hypothesis *H*_1_ of the study is supported. Furthermore, no statistically significant direct effect of servant leadership on quiet quitting has been identified (*b* = −0.062, *p* > 0.05). However, the interaction effect between job creep and servant leadership on quiet quitting is found to be statistically significant (*b* = 0.129, *p* < 0.05). In line with these findings, the slope analysis graph illustrating the moderating effect is presented in Figure 5.

An examination of the moderating effect illustrated in Figure 5 reveals that the impact of job creep on quiet quitting becomes progressively stronger at low (*b* = 0.731, *p* < 0.001), moderate (*b* = 0.842, *p* < 0.001), and high (*b* = 0.953, *p* < 0.001) levels of servant leadership. As employees’ perceptions of servant leadership increase, the positive effect of job creep on quiet quitting is amplified. These findings indicate that servant leadership moderates the relationship between job creep and quiet quitting. Accordingly, Hypothesis *H*_2_ of the study is supported.

## 5 Discussion

This study examines the effect of job creep on quiet quitting and the moderating role of servant leadership in this relationship. The findings show that job creep significantly and positively influences employees’ quiet quitting behavior. This result reveals that tasks exceeding formal job descriptions can become hidden obligations over time, reducing employee motivation and increasing withdrawal and silencing behaviors. According to the findings, servant leadership does not have a direct significant effect on quiet quitting. However, servant leadership plays a significant moderating role in the relationship between job creep and quiet quitting. As employees’ perceptions of servant leadership increase, the effect of job creep on quiet quitting is strengthened. This finding shows that servant leadership cannot always play a supportive role for employees; under certain conditions, it can cause employees to internalize an increasing workload. This situation reveals that the supportive and empathetic approaches of servant leaders will strongly resonate with employees. Over time, this can normalize expectations beyond employees’ main tasks, causing emotional burnout and psychological withdrawal. This study investigated the effect of job creep on quiet quitting and the moderating role of servant leadership in this relationship. The findings indicate that job creep is positively and significantly associated with quiet quitting. This result suggests that role expansions that occur outside formal job descriptions can be defined as organizational job demands that contribute to the emergence of silencing behaviors among employees. Servant leadership did not have a direct significant effect on quiet quitting. However, servant leadership played a significant moderating role in this relationship. Specifically, the findings indicate that the positive relationship between job creep and quiet quitting is stronger when employees perceive servant leadership to be high. This finding indicates that servant leadership may not always fulfill its protective function regarding job-related resources, and that under certain conditions, unexpected consequences may occur. This result reveals that servant leadership strengthens employees’ moral obligation and emotional commitment. While this is considered a positive outcome, it can lead to a normalization of increased workload by raising employees’ tendency to accept additional responsibilities outside their main duties over time. Over time, this situation can lead to resource depletion and psychological withdrawal. This finding aligns with studies highlighting the “dark side” of trans-role behaviors and excessive organizational expectations (Bolino et al., 2013) and with the organizational behavior literature suggesting that high levels of social support or benevolent leadership can sometimes create a ‘commitment trap’ or performance anxiety in high-stress environments (e.g., Güler, 2025).

## 6 Conclusion and implications

### 6.1 Theoretical implications

This study contributes to the Job Demands-Resources (JD-R) theory by positioning job overflow as a significant job demand and considering it an explanatory factor in the emergence of quiet quitting. It also contributes a new perspective to the literature by demonstrating that servant leadership may not always function as a job resource mitigating the negative effects of job demands. Furthermore, because few studies address the relationship between job overflow and quiet quitting and examine the moderating role of servant leadership in that relationship, this study fills a significant gap in the organizational behavior literature. This study offers three key contributions to the Job Demands-Resources literature. First, it expands the job demands literature by positioning job overflow as a chronic role expansion beyond formal duties. Second, it adds a previously underexamined variable to the literature on quiet quitting. Third, it reveals that the impact of job resources may vary depending on circumstances, demonstrating that servant leadership may not always serve a supportive role within the Job Demands-Resources framework.

### 6.2 Practical implications

The research findings highlight the importance of managers clearly defining employee roles and responsibilities. Otherwise, normalizing expectations that extend beyond core roles over time can increase psychological withdrawal among employees. Furthermore, managers must take care, when implementing supportive leadership practices, not to encourage employees to internalize excessive workloads. A balanced application of the fundamental characteristics of servant leadership, such as empathy, support, and fairness, is crucial. Organizations should also develop open communication channels that allow employees to report workload and stress experiences. The findings have important implications for managers and organizations. First, clearly defining job roles and responsibilities is critical to preventing the normalization of expectations beyond one’s role over time. Otherwise, this can lead to increased psychological withdrawal among employees.

Second, while servant leadership is generally considered a positive leadership approach, it can have unexpected effects in environments with increasing work demands.

Overly empathetic approaches, emphasis on managerial support, and self-sacrifice can reinforce employees’ desires and perceptions of taking on more responsibility. Thirdly, it is important for organizations to develop robust and effective employee communication systems that enable employees to report increased workload and psychological distress promptly and comfortably. Such systems can prevent work overload from turning into quiet quitting. Recommendations for practitioners are as follows:

- Employees’ job descriptions should be clearly defined, and systematization of non-job-related tasks should be avoided.
- The workload should be distributed fairly and evenly.
- Employees’ performance should be evaluated solely based on their job-related responsibilities.
- While adopting a servant leadership approach, leaders should take measures to avoid indirectly burdening employees with excessive responsibilities.
- Managers should be able to identify early signs of employee burnout and quiet quitting.

## 7 Limitations and recommendations

Although servant leadership is conceptualized as a resource within the framework of Job Demands-Resources (JD-R) theory, its moderating role in the relationship between increased workload and employees’ silent disengagement may produce effects that are more complex than merely supportive. Recent developments in leadership research indicate that employee-centered and communication-focused leadership styles can produce adverse psychological consequences under conditions of increasing job demands. In particular, servant leadership can strengthen employees’ feelings of moral obligation and indebtedness to the leader and the organization. Although these results are positively received, they can also increase employees’ willingness to accept additional responsibilities beyond their formal role descriptions. In situations where role expectations increase and become normalized, employees may be less likely to resist increased demands. In this case, employees may accept non-role expectations as part of their core roles, leading to the depletion of resources over time. From a job demands-resources perspective, this shows that job resources not only help fulfill job demands but can also lead to negative consequences as demands increase. In such cases, servant leadership can inadvertently reinforce the link between increased workload and quiet quitting by fostering excessive commitment.

This study has some limitations. Firstly, limiting the research to a specific sector and region (manufacturing sector, Karaman Organized Industrial Zone) reduces the generalizability of the findings. It is recommended that future research be replicated across different sectors with larger samples. Second, because the study employs a cross-sectional design, definitive causal inferences about relationships between variables cannot be made. It is recommended that longitudinal designs be used in future studies. In addition, this study only examined the moderating role of servant leadership. It is recommended that mediating and moderating variables, such as psychological empowerment, organizational justice, and leader-member interaction, be included in the model for future research. Finally, quiet quitting can manifest in different forms at the individual and organizational levels. Therefore, employing multi-level and qualitative research designs in future studies may contribute to a deeper understanding of the phenomenon. The primary rationale for choosing a cross-sectional design in this study is its time- and cost-effectiveness, allowing data collection from a large sample of employees in manufacturing businesses in the Karaman Organized Industrial Zone over a short period and within defined margins of error and confidence limits. Furthermore, the cross-sectional approach is suitable for identifying the current state of dynamic organizational variables such as job creep, quiet quitting, and servant leadership, and the regulatory relationships between them, based on a snapshot in time.

## 8 Conclusion

This study reveals that job creep is a significant determinant of quiet quitting and that this relationship can vary depending on perceptions of servant leadership. The findings, analyzed within the framework of Job Demands-Resources Theory, indicate that the role of job demands and job resources in shaping employee behavior requires re-evaluation. Overall, the results show that clarifying job design and descriptions, having clear role definitions, and implementing leadership practices in a balanced way to support employees play a critical role in the psychological well-being of employees.

## Data Availability

The original contributions presented in the study are included in the article/supplementary material, further inquiries can be directed to the corresponding author.
